# Cost-Effectiveness of Conbercept vs. Ranibizumab for Age-Related Macular Degeneration, Diabetic Macular Edema, and Pathological Myopia: Population-Based Cohort Study and Markov Model

**DOI:** 10.3389/fmed.2021.750132

**Published:** 2021-12-02

**Authors:** Zhuang Cui, Wei Zhou, Qinxue Chang, Tiantian Zhang, Hui Wang, Xiangda Meng, Yuanyuan Liu, Hua Yan

**Affiliations:** ^1^Department of Epidemiology and Biostatistics, School of Public Health, Tianjin Medical University, Tianjin, China; ^2^Department of Ophthalmology, Tianjin Medical University General Hospital, Tianjin, China

**Keywords:** age-related macular degeneration (AMD), diabetic macular edema (DME), pathological myopia (PM), ranibizumab, conbercept, cost-effectiveness, Markov model

## Abstract

**Background:** With the advent of aging society of China, fundus diseases related to pathological neovascularization, including age-related macular degeneration (AMD), diabetic macular edema (DME), and pathological myopia (PM), have become an increasingly serious medical and health problems. As effective drugs of the treatment, conbercept and ranibizumab have been commonly used and covered by the national basic medical insurance in China. However, the pharmacoeconomic evaluation of conbercept vs. ranibizumab for DME and PM remains lacking. This study would assess the cost-effectiveness of conbercept and ranibizumab for the treatment of AMD, DME, and PM from the perspective of Chinese payers.

**Methods:** A Markov chain model was constructed based on the visual conditions of the patient indicated by the number of letters in best corrected visual acuity (BCVA). We conducted models based on real-world scenario to calculate the cost per the quality-adjusted life-year (QALY) gained. A 1-year cycle length and a 10-year simulation treatment were applied and the number of injections of conbercept and ranibizumab was assumed to the average number within 10 years. Transition probabilities, costs, utility data, and other parameters were obtained from literature searches. A 3.5% discounting rate was applied for both the costs and utilities.

**Results:** The incremental cost-effectiveness ratios (ICERs) were more favorable for conbercept than ranibizumab in treatment of AMD, DME, and PM, with associated ICER of 66,669 renminbi (RMB), −258,813 RMB, and −373,185 RMB per QALY gained. Compared with ranibizumab, the incremental effectiveness of conbercept in treatment of AMD, DME, and PM was −0.665 QALYs, 0.215 QALYs, and 0.029 QALYs, respectively. The sensitivity analysis showed the same findings, although the ICER is sensitive to the costs of this program.

**Conclusion:** Under the current Chinese healthcare setting, conbercept is suitable and cost-effective in treatment of AMD, DME, and PM compared with ranibizumab.

## Introduction

Age-related macular degeneration (AMD), a chronic macular disease that affects the central retina, is the third leading cause of irreversible blindness and the first cause of visual impairment in developed countries (1–3). The total number of patients with AMD was estimated to be 196 million in 2020 and might rise to 288 million by 2040 (4). Global burden of disease in 2010 showed that vision-related quality-adjusted life-years (QALYs) caused by AMD increased at an exponential rate of 160% (5). Diabetic retinopathy is the leading blinding eye disease for working-aged people and diabetic macular edema (DME) secondary to diabetic retinopathy is the direct cause of visual impairment (6). Epidemiological surveys in China show that the incidence of DME in diabetic patients is 5% (7, 8). Pathological myopia (PM) is one of the three most common causes of blindness in the world. As many as 3% of the population of the world suffer from PM, especially in Asian countries, and the global prevalence is generally increasing (9). Thus, the visual impairment caused by fundus diseases related to pathological neovascularization, including AMD, DME, and PM, has brought a heavy economic burden to the society.

In China, with the advent of an aging society, AMD, DME, and PM have become an increasingly serious medical and health problems, for which intravitreal injection of antivascular endothelial growth factor (VEGF) drugs is an effective method. Until now, the main anti-VEGF drugs in clinical use are ranibizumab, aflibercept, bevacizumab, and conbercept, which have been proven to be superior to conventional treatments (10).

Ranibizumab, the first drug proven to improve eyesight, has been confirmed that it has good effects through randomized controlled trials (RCTs) (11–13). Conbercept (Lumitin, Chengdu Kanghong Biotech Corporation Ltd., Chengdu, Sichuan, China), a genetically engineered fusion protein, is an anti-VEGF drug independently developed by China in the recent years. It can effectively combine with VEGF in blood vessels and tissues to treat a variety of eye diseases related to pathological angiogenesis (14). A recent systematic review demonstrated that conbercept had comparable safety and efficacy profiles rather than ranibizumab (15). Ranibizumab and conbercept have been commonly used and covered by the national basic medical insurance in China, so it is necessary to conduct an economic evaluation of these two drugs, which socioeconomic burden would be fueled soon for their high cost and widespread used. An economic evaluation has concluded previously that conbercept is more cost-effective than aflibercept and ranibizumab for the treatment of AMD in China, but only based on the 2-year medication situation in clinical scenarios (16). Even worse, there is still a lack of studies on the pharmacoeconomic evaluation between conbercept and ranibizumab treatment of DME and PM. Therefore, it is urgent to evaluate the cost-effectiveness between conbercept and ranibizumab in treatment of AMD, DME, and PM from the perspective of Chinese payers in order to optimally allocate and utilize the limited medical resources.

## Methods

A Markov chain model, the simplest Markov model, was used to estimate the cost-effectiveness of conbercept and ranibizumab in treatment of AMD, DME, and PM. The principle is to simulate the development of the disease according to the transition probability of its natural process, divided into different states (Markov state) in a certain period of time (Markov cycle). Combined with the health utility value of the disease and the consumption of public health resources, the outcome and cost of disease progression were estimated through multiple iterations.

**Figure 1:**
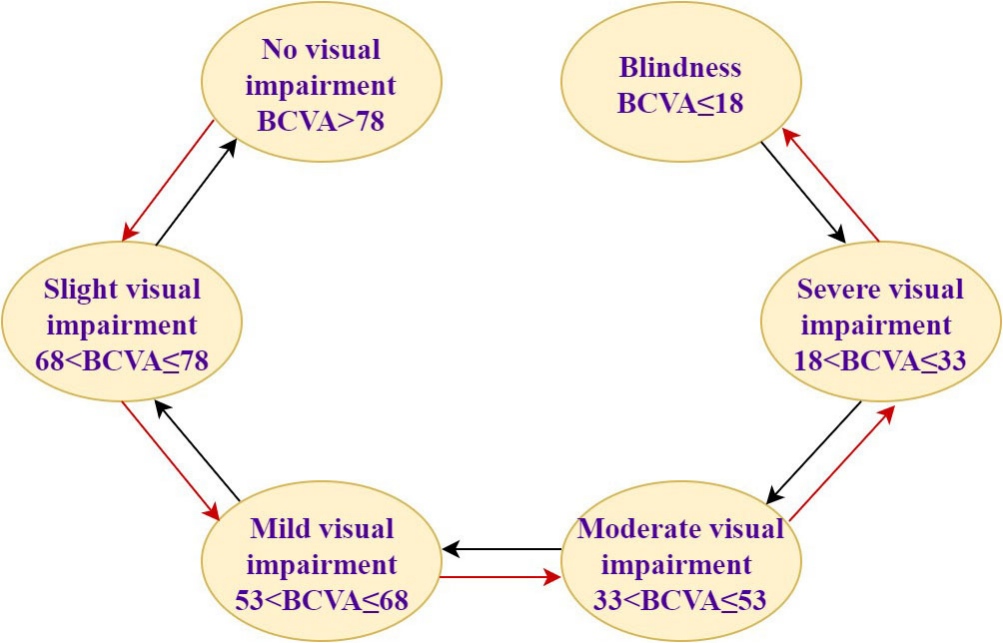
The Markov model structure of age-related macular degeneration.

**Figure 2:**
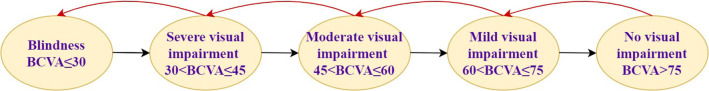
The Markov model structure of diabetic macular edema.

**Figure 3:**
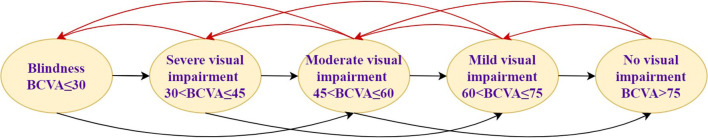
The Markov model structure of pathological myopia.

In this study, assuming that the simulated population is patients with AMD, DME, and PM, the baseline characteristics are consistent with that in PHOENIX (identifier NCT01436864), SAILING (identifier NCT02194634), and SHINY (identifier NCT01809223) studies, respectively (Appendix Part A). Health states were represented by different vision conditions of the patients, which were indicated by the number of letters in best corrected visual acuity (BCVA). There are six health states of AMD and five health states of the other two eye diseases. The specific health status classification and baseline visual acuity distribution of the patient are shown in Table 1. The baseline distribution of visual acuity among the patients with AMD, DME, and PM was derived from unpublished data in PHOENIX, SAILING, and SHINY studies, respectively. The average annual number of injections was used to simulate the injection situation within 10 years, assuming that remained the same. The number of injections for conbercept and ranibizumab was set according to the existing literature and research. The difference between the costs of the two drugs has remained approximately the same within 10 years due to focus on the incremental cost-effectiveness ratio (ICER) in this study, which is the ratio of incremental cost to incremental effectiveness. The ICER is used to evaluate the relative economics of two or more alternative treatment options. The Markov transition processes of the eye diseases were shown in Figures 1–3, respectively, and the arrows indicate whole possible transitions between the states 1 year later.

**Table 1:** The health status classification and baseline visual acuity distribution.

	**Age-related macular degeneration**		**Diabetic macular edema**		**Pathological myopia**	
No visual impairment	BCVA > 78	/	BCVA > 75	/	BCVA > 75	/
Slight visual impairment	68 < BCVA ≤ 78	11.11%	-	-	-	-
Mild visual impairment	53 < BCVA ≤ 68	38.27%	60 < BCVA ≤ 75	43.6%	60 < BCVA ≤ 75	29.38%
Moderate visual impairment	33 < BCVA ≤ 53	32.10%	45 < BCVA ≤ 60	41.9%	45 < BCVA ≤ 60	44.63%
Severe visual impairment	18 < BCVA ≤ 33	17.28%	30 < BCVA ≤ 45	11.7%	30 < BCVA ≤ 45	20.34%
Blindness	BCVA ≤ 18	1.23%	BCVA ≤ 30	2.8%	BCVA ≤ 30	5.65%
Reference	the PHOENIX study		the SAILING study		the SHINY study	

After a cycle, participants in one stage could stay in that stage or move onto the next stage with a certain transition probability. Suppose that after 1 year of the treatment, there were three types of therapeutic effects of AMD and DME: BCVA increased by 15 letters or more, BCVA remained unchanged, BCVA decreased by 10 letters or more, five types of therapeutic effects of PM: BCVA increased or decreased by 30 letters, BCVA increased or decreased by 15 letters, and BCVA remained unchanged. The model structure is shown in Figures 1–3.

## Input Parameters

### Transition Probabilities

The conditional probability of a given stage moving to another stage after a certain time is expressed by transition probabilities. The metastasis probability table of AMD, DME, and PM is shown in Table 2, which represented the conditional probability of transition from a given stage to another stage after 1 year. The transition probability of this study was obtained from the related clinical trial literature to simulate the real-world treatment situation.

**Table 2:** Best corrected visual acuity (BCVA) transition probabilities in 0–12 months.

**BCVA change**	**Conbercept**	**Ranibizumab**
**Age-related macular degeneration**		
15	32.10%	43.00%
0	50.62%	48.2%
−15	17.28%	8.80%
Reference	(17)	(13)
**Diabetic macular edema**		
15	25.00%	18.50%
0	72.32%	78.5%
−15	2.68%	3.00%
Reference	(18)	(19)
**Pathological myopia**		
30	18.32%	20.03%
15	53.44%	49.13%
0	25.19%	27.55%
−15	3.05%	3.20%
−30	0.00%	0.07%
Reference	(20)	(20, 21)

### Utilities

The generic health status and health-related quality of life were expressed by health utility weights. According to how the individuals perceive his or her health status, health utility weights are valued between 0 and 1. Utility of 1 was considered as “perfect health,” while utility of 0 was considered as “death.” AMD, DME, and PM can be monitored by changes in visual acuity (VA) and VA results are presented using the logarithm of the minimum angle of resolution (logMAR) scale. A previous study performed the univariate ordinary least squares regression analyses to investigate the strength of the relationship between VA and the utility value obtained from time trade-off of the patients and found that the utility value has a linear relationship with VA, which manifested as the utility value equals 0.828–0.359 × logMAR (22). We used this formula to estimate the health utility value of DME and PM under different letter states due to the difficulty of obtaining them. Utility values of AMD obtained from the literature are included in Table 3.

**Table 3:** Health utility value.

	**Age-related** **macular** **degeneration**	**Diabetic** **macular** **edema**	**Pathological** **myopia**
No visual impairment	0.9	0.7562	0.7562
Slight visual impairment	0.85	-	-
Mild visual impairment	0.81	0.6485	0.6485
Moderate visual impairment	0.57	0.5408	0.5408
Severe visual impairment	0.52	0.4331	0.4331
Blindness	0.4	0.3254	0.3254
Reference	(23, 24)	(22)	(20, 22)

### Costs

In this study, costs for patient management were considered as direct medical costs (including drug costs, inspection costs, surgical costs, nursing costs, and treatment costs) for efficacy end point events. The prices of single injections of conbercept and ranibizumab were 5,550 RMB and 5,700 RMB, respectively, according to the National Reimbursement Drug List (NRDL) (2019). A 1-year drug treatment cycle was adopted in our analysis. The injections of conbercept and ranibizumab were used as average annual number within 10 years to investigate the influence of the variance on cost estimates. Both the cost and the health utility value used a discount rate of 3.5% (Table 4).

**Table 4:** The number of injections of conbercept and ranibizumab in real-world scenario.

**Parameters**	**Age-related** **macular** **degeneration**	**Diabetic** **macular** **edema**	**Pathological** **myopia**
**Conbercept**			
Average annual number of injections	4.8	5.5	1.8^*^
Reference	(25–27)	(28–32)	(33)
**Ranibizumab**			
Average annual number of injections	5.4	6.2	1.92^*^
Reference	(34–40)	(32, 41–45)	(46)

* *The number of injections in 1st year in the real-world setting in China*.

### Sensitivity Analysis

Because the decisions informed by the model are affected by parameter uncertainty, it is necessary to carry out extensive sensitivity analyses including the fluctuation of drug prices and different numbers of injections to test the robustness of the results. First, we have compared the cost-effectiveness of treating AMD, DME, and PM when the prices of conbercept and ranibizumab fluctuate by 5 and 10%. Moreover, we have compared the cost-effectiveness of conbercept and ranibizumab at latest negotiated prices that are 4,160 RMB and 3,950 RMB, respectively, since January 1, 2020 to December 31, 2021, according to the NRDL (2020). Second, we have carried out one-way and two-way sensitivity analysis on the number of injections of conbercept and ranibizumab based on the number of drug injections in real world in the existing literatures. Third, we assumed that the cost obeyed the gamma distribution in probabilistic sensitivity analysis using Monte Carlo simulation (47). The cost-effectiveness acceptability curves of conbercept and ranibizumab in treatment of AMD, DME, and PM are shown in Appendix Part B. Finally, the number of injections in the real world is often smaller than that in RCT, so the cost-effectiveness analysis of different injections in RCT scenario is also part of the sensitivity analysis. The number of injections in RCT scenario was derived from previous studies (Table 5).

**Table 5:** Sensitivity analyses about number of injections of conbercept and ranibizumab in randomized controlled trial (RCT) scenario.

**Parameters**	**Age-related** **macular** **degeneration**	**Diabetic** **macular** **edema**	**Pathological** **myopia**
**Conbercept**			
Number of injections in 1st year	5.8	9.48	3.76
Reference	(17)	(18)	(48)
**Ranibizumab**			
Number of injections in 1st year	11.5	7.9	3.9
Reference	(13)	(19)	(21)

### Willingness to Pay

In developing countries, 3-fold the per capita gross domestic product (GDP) is usually taken as the maximum WTP (33). In 2019, GDP per capita of China was 70,892 RMB. The maximum WTP for a QALY gained in this program of about 212,676 RMB was estimated based on the above approach.

## Results

### Cost-Effectiveness Comparison of Conbercept and Ranibizumab in Real-World Scenario

The costs of conbercept and ranibizumab for AMD, DME, and PM within 10 years in real-world scenario are shown in Table 6.

**Table 6:** The cost-effectiveness analyses of conbercept and ranibizumab in real-world scenario.

	**Strategy**	**Cost (RMB)**	**Incremental costs**	**Effectiveness** **(QALYs)**	**Incremental** **effectiveness**	**CER**	**ICER**
Age-related macular degeneration	Conbercept	285,285.597	−44,334.924	7.825	−0.665	36,458.223	66,669.059
	Ranibizumab	329,620.521	-	8.490	-	38,824.561	-
Diabetic macular edema	Conbercept	322,763.734	−55,689.457	6.973	0.215	46,285.914	−258,813.897
	Ranibizumab	378,453.191	-	6.758	-	56,000.033	-
Pathological myopia	Conbercept	106,587.011	−10,611.396	7.528	0.029	14,159.467	−373,185.397
	Ranibizumab	117,198.407	-	7.499	-	15,628.163	-

For ranibizumab, the costs were 329,620 RMB, 378,453 RMB, and 117,198 RMB in treatment of AMD, DME, and PM within 10 years, respectively, while the costs of conbercept were 285,285 RMB, 322,763 RMB, and 106,587 RMB, respectively. Compared with ranibizumab, the incremental costs of conbercept were −44,334 RMB, −55,689 RMB, and −10,611 RMB, respectively, and the ICER were 66,669 RMB, −258,813 RMB, and −373,185 RMB per QALY gained, less than the threshold of triple GDP per capita. Conbercept had significant cost-effectiveness in treatment of AMD, DME, and PM because ranibizumab was dominated by conbercept, meaning that it was more costly and less effective.

### Cost-Effectiveness Comparison of Conbercept and Ranibizumab in RCT Scenario

The treatment costs of conbercept and ranibizumab for AMD, DME, and PM within 10 years in RCT scenario are shown in Table 7.

**Table 7:** The cost-effectiveness analyses of conbercept and ranibizumab in RCT scenario.

	**Strategy**	**Cost (RMB)**	**Incremental costs**	**Effectiveness (QALYs)**	**Incremental effectiveness**	**CER**	**ICER**
Age-related macular degeneration	Conbercept	34,0799.706	−361,169.923	7.825	−0.664	43,550.234	543,719.770
	Ranibizumab	701,969.628	-	8.490	-	82,684.890	-
Diabetic macular edema	Conbercept	556,327.309	74,104.695	6.973	0.215	79,780.084	344,397.772
	Ranibizumab	482,222.614	-	6.758	-	71,354.881	-
Pathological myopia	Conbercept	222,648.423	−15,410.842	7.528	0.029	29,577.553	−541,974.025
	Ranibizumab	238,059.265	-	7.499	-	31,744.706	-

The costs of ranibizumab were 70,1969 RMB, 482,222 RMB, and 238,059 RMB for AMD, DME, and PM within 10 years, respectively, while the costs of conbercept were 340,799 RMB, 556,327 RMB, and 222,648 RMB, respectively. Compared with ranibizumab, the incremental costs of conbercept were−361,169 RMB, 74,104 RMB, and−15,410 RMB, respectively. The ICER was−54,1974 RMB per QALY gained for PM, with much less than the threshold of triple GDP per capita and ranibizumab cost twice as much as conbercept for AMD with a similar QALY, which means that compared with ranibizumab, conbercept had cost-effectiveness in treatment of AMD and PM in RCT scenario.

### Extensive Sensitivity Analyses

The sensitivity analyses have indicated that at latest negotiated prices, conbercept still has a significant cost-effectiveness in treatment of AMD, DME, and PM compared with ranibizumab. When the price remains and the number of drug injections changes, conbercept has a certain cost-effectiveness in treatment of AMD with similar number of injections (Appendix Part B).

## Discussion

To the best of our knowledge, this is the first study to analyze and compare the cost-effectiveness of conbercept and ranibizumab for the treatment of AMD, DME, and PM based on the real-world scenarios. The results of the economic evaluation study suggest that compared with ranibizumab, conbercept has lower cost and more effective in treatment of DME and PM, which indicated that conbercept had significant cost-effectiveness. In treatment of AMD, the total cost and total utility value of conbercept were lower than that of ranibizumab within 10 years, while the ICER was lower than the threshold of three times per capita GDP. The results indicated that conbercept had a certain cost-effectiveness as well relative to ranibizumab in treatment of AMD.

Previous cost-effectiveness study based on the status change of BCVA, using a similar Markov structure, has explored the cost-effectiveness of conbercept and ranibizumab for wet AMD and found that conbercept was a more cost-effective option for the treatment of AMD in a Chinese healthcare setting (16). A meeting abstract from the International Society for Pharmacoeconomics and Outcomes Research (ISPOR) also mentioned that compared with ranibizumab, conbercept is a superior cost-saving alternative for the treatment of Chinese AMD (49). Additionally, conbercept also has certain advantages in clinical application. Unlike ranibizumab, a recombinant humanized anti-VEGF monoclonal antibody Fab fragment, which can only neutralize all the active components of VEGF-A, conbercept is a recombinant fusion protein of the extracellular segments of VEGF receptors 1 and 2 and the human immunoglobulin Fc segment, which can target VEGF-A, VEGF-B, and placental growth factor (15, 50). Therefore, among the four anti-VEGF drugs such as ranibizumab, aflibercept, bevacizumab, and conbercept, conbercept is a 100% humanized protein with the highest affinity and the most binding VEGF targets, which avoids the immune risk of murine protein. The high affinity also means a longer active period of the drug, thereby extending the treatment interval, reducing the burden of the patient, and reflecting the therapeutic advantage in AMD, DME, and PM.

Age-related macular degeneration, induced by a variety of factors and closely related to age, can occur in middle age and its incidence shows an exponential increase after the age of 70 years (51). At the same time, it has been estimated that 25% of Asians will be aged over 60 years by 2050 (52). Therefore, with aging global populations, it was anticipated that AMD would continue to be major causes of vision impairment (4). High myopia is particularly prevalent in East Asian populations and the prevalence of high myopia in the Chinese population in Singapore is 9.1%, China is 3.3%, Taiwan is 2.4%, and Japan is 8.2% (53, 54). PM can develop from high myopia, brings severe visual impairment, and its prevalence is relatively high (about 9–21%) in adults in Asian populations (55). DME is a diabetic microvascular complication that occurs in the retina (56) and diabetic retinopathy (DR) is a common diabetic complication and the main cause of moderate-to-severe visual impairment in diabetic patients (57). About one-third of diabetic patients develop DR and about one-third of patients with DR develop DME (58). Until now, diabetes mellitus (DM) remains a major health burden, with ~80% occurring in low- and middle-income countries (59, 60). The number of DM in China is expected to increase to 142.7 million by 2035 (61). Similarly, it was predicted that the diabetic population in Malaysia would continue to rise (62).

In summary, in low- and middle-income countries that have many similarities and comparability with China in terms of national conditions, such as Malaysia and Singapore, the disease burden of AMD, DME, and PM is also heavy. Although the European Society of Retina Specialists recommends anti-VEGF therapy as a first-line treatment, it is unclear which anti-VEGF should be used first (63). Economic evaluation and analysis should be carried out to obtain greater benefits at the minimum cost and then optimize the allocation and utilization of limited medical resources. This study was based on real-world data for long-term evaluation, which is objective, scientific, and authentic, and can reflect the medication and health of the patient under real conditions to the maximum extent. Therefore, the conclusion throws significant light on the treatment of AMD, DME, and PM in low- and middle-income countries such as Malaysia.

Our research findings showed that in order to maximize health within the fixed general healthcare budget, in the case of the equivalence of conbercept and ranibizumab, the preferential price of conbercept enables it to achieve good therapeutic effect and saves the medical cost during treatment, which has certain advantages. Therefore, clinicians could consider using conbercept instead of ranibizumab to treat AMD, DME, and PM.

This analysis has some limitations that need to be considered along with the results. Firstly, this study mainly used parameters extracted from foreign reports and articles, which might cause a certain degree of uncertainty, so we carried out extensive sensitivity analyses to prove the credibility and robustness of our research results. Secondly, there were more or less differences in population characteristics under different research backgrounds, which may cause the results to have a certain orientation and lack the ability to generalize. Moreover, indirect costs such as the loss of productivity and adverse effects of conbercept and ranibizumab were not included in this study, which may cause a heavy socioeconomic burden for the family and society of the patient. According to clinical trials, the incidence of adverse effects of the two treatment options is very low and mild; the cost-effectiveness of conbercept vs. ranibizumab would become more credible, if this study included the indirect costs. Finally, we simply compared the cost-effectiveness of conbercept and ranibizumab. In fact, compared with other potential anti-VEGF therapies (aflibercept and bevacizumab), ranibizumab has not been shown to be cost-effective in treatment of AMD, DME, and PM (64–66). In light of fact that bevacizumab has been admitted to medical insurance in China in 2017, it is necessary to conduct the cost-effectiveness of conbercept vs. aflibercept or bevacizumab in follow-up studies.

## Conclusion

In conclusion, from the third-party payer perspective, conbercept was more cost-effective than ranibizumab in treatment of AMD, DME, and PM. Therefore, due to its favorable economic outcomes, conbercept was the most suitable option in the current Chinese healthcare setting, although conbercept and ranibizumab were both licensed for the treatment of the three eye diseases in China.

## Data Availability Statement

The original contributions presented in the study are included in the article/Supplementary Material, further inquiries can be directed to the corresponding author.

## Author Contributions

ZC and WZ collected data. TZ, HW, and QC designed the entire plan of this study and wrote the manuscript. XM and YL did data analysis and interpretation. ZC, WZ, and HY discussed and supervised this study. All the authors wrote the manuscript and approval the final manuscript.

## Funding

This study was supported by the National Natural Science Foundation of China (Grant Numbers 82020108007 and 81830026) and the Beijing-Tianjin-Hebei Special Project (Grant Numbers 19JCZDJC64300(Z) and 20JCZXJC00180).

## Conflict of Interest

The authors declare that the research was conducted in the absence of any commercial or financial relationships that could be construed as a potential conflict of interest.

## Publisher's Note

All claims expressed in this article are solely those of the authors and do not necessarily represent those of their affiliated organizations, or those of the publisher, the editors and the reviewers. Any product that may be evaluated in this article, or claim that may be made by its manufacturer, is not guaranteed or endorsed by the publisher.
