# Supplementary material for: Cost-Effectiveness of Conbercept vs. Ranibizumab for Age-Related Macular Degeneration, Diabetic Macular Edema, and Pathological Myopia: Population-Based Cohort Study and Markov Model
Source: Front Med (Lausanne). 2021 Dec 2;8:750132. doi: 10.3389/fmed.2021.750132 (PMC8676057; doi:10.3389/fmed.2021.750132)
Supplement: Supplementary file 1 [file Data_Sheet_1.docx]

**Part A. The Supplementary Information of the PHOENIX, SAILING and SHINY studies**

**Table S1** The health status classification and baseline visual acuity distribution

|  | the PHOENIX study (n=81) | | | the SAILING study (n=248) | | | the SHINY study (n=177) * | | |
| --- | --- | --- | --- | --- | --- | --- | --- | --- | --- |
| No visual impairment | BCVA > 78 | / | / | BCVA > 75 | / | / | BCVA > 75 | / | / |
| Slight visual impairment | 68< BCVA≤ 78 | 9 | 11.11% | - | - | - | - | - | - |
| Mild visual impairment | 53< BCVA≤ 68 | 31 | 38.27% | 60< BCVA≤ 75 | 108 | 43.6% | 60< BCVA≤ 75 | 52 | 29.38% |
| Moderate visual impairment | 33< BCVA≤ 53 | 26 | 32.10% | 45< BCVA≤ 60 | 104 | 41.9% | 45< BCVA≤ 60 | 79 | 44.63% |
| Severe visual impairment | 18< BCVA≤ 33 | 14 | 17.28% | 30< BCVA≤ 45 | 29 | 11.7% | 30< BCVA≤ 45 | 36 | 20.34% |
| Blindness | BCVA ≤ 18 | 1 | 1.23% | BCVA ≤ 30 | 7 | 2.8% | BCVA ≤ 30 | 10 | 5.65% |
| The data in the table are the number and percentage of patients with corresponding baseline visual acuity.  * The number of participants in the SHINY study was 176 when designed, and 177 participants were actually enrolled. | | | | | | | | | |

The PHOENIX Study

**Study Design**

**Study Type:** Interventional (Clinical Trial)

**Actual Enrollment:** 125 participants

**Allocation:** Randomized

**Intervention Model:** Parallel Assignment

**Masking:** Quadruple (Participant, Care Provider, Investigator, Outcomes Assessor)

**Primary Purpose:** Treatment

**Official Title**: A Phase 3 Clinical Trial of Intravitreal Injections of Human Recombinant Vascular Endothelial Growth Factor Receptor-Fc Fusion Protein in Patients With Choroidal Neovascularization Secondary to Age-related Macular Degeneration.

**Intervention:** Recombinant Human VEGF Receptor-Fc Fusion Protein (Intravitreal injection of KH902 once per month)

**Study Arms：**

- Experimental: 0.5 mg KH902

Patients will receive intravitreal injection of KH902 0.5mg/eye once per month for three times in the study eye, and then patients will receive 2 sham injections monthly, respectively, at the end of month 3 (visit 5), and following these injections you will receive intravitreal injection of KH902 once every three months, till month 12 (respectively at month 5, month 8 and month 11)

**Intervention:** Biological: Recombinant Human VEGF Receptor-Fc Fusion Protein

- Sham Comparator: Sham-injection

Patients will receive sham injection once per month for three times, and then you will receive intravitreal injection of KH902 0.5mg/eye once per month for three times in the study eye, after three months' treatment they will receive intravitreal injection of KH902 once every three months, till month 12 (respectively at month 8 and month 11).

**Intervention:** Biological: Recombinant Human VEGF Receptor-Fc Fusion Protein

**Eligibility Criteria**

**Ages Eligible for Study:** 50 Years and older (Adult, Older Adult)

**Sexes Eligible for Study:** All

**Accepts Healthy Volunteers:** No

**Criteria:**

1. Inclusion Criteria:

- Signed the Informed Consent Form;
- Age ≥ 50 years of either gender;
- Total lesion size ≤ 30 mm2 of the study eye;
- BCVA score of the study eye between 73 and 19 letters;
- Clear ocular media and adequate pupil dilation to permit good quality fundus photographic imaging.
- BCVA score of the fellow eye ≥ 19 letters.

1. Exclusion Criteria:

- Current or previous non-exudative AMD diseases which affect the inspection and measurement of macular or the central visual acuity;
- Subretinal hemorrhage area≥ 50% of total lesion size;
- Scar or fibrosis area in study eyes ≥ 50% of total lesion size; or central foveal scar、fibrosis or atrophy of macular in the study eye;
- Presence of retinal pigment epithelial tear, retinal macular tractional, macular epiretinal membrane, and diagnosed with polypoidal choroidal vasculopathy in the study eye;
- Previous anti-VEGF drug treatment in the study eye within six months preceding screening; or anti-VEGF treatment in the fellow eye within three months before screening;
- Previous intraocular or periocular operations, excluding operations on eyelid without hampering the intravitreal injection in the study eye;
- Previous ophthalmologic operations in the study eye;
- Current active inflammation or infection in either eye;
- Uncontrolled previous or current glaucoma in either eye, or previous glaucoma filtering operation in the study eye;
- Current systemic administrations which may lead to toxicity in the crystalline lens;
- History of allergy or current allergic response;
- History of surgery within one month preceding enrollment;
- Infectious diseases need systemic administration;
- Systemic autoimmune diseases;
- Any uncontrolled clinical disorders;
- Patients of child-bearing potential do not adopted adequate contraception methods;
- Pregnant or nursing women;
- Patients should be excluded in the opinion of investigators;

The SAILING Study

**Study Design**

**Study Type:** Interventional (Clinical Trial)

**Actual Enrollment:** 248 participants

**Allocation:** Randomized

**Intervention Model:** Parallel Assignment

**Masking:** Quadruple (Participant, Care Provider, Investigator, Outcomes Assessor)

**Primary Purpose:** Treatment

**Intervention:**

- Drug: Conbercept (Intravitreal injection of 0.5 mg Conbercept at first month, then repeated as needed.)
- Other: Sham injection (Sham intravitreal injection at first month, then repeated as needed.)
- Procedure: Laser (Laser treatment at first month, then repeated as needed.)
- Other: Sham laser (Sham laser at first month, then repeated as needed.)

**Study Arms:**

- Experimental: Conbercept treatment group

Conbercept injection and sham laser treatment at day 0 for 1st time, the investigators will decide whether the subjects need to get repeated treatment according to monthly assessment.

**Interventions:**

- - Drug: Conbercept
  - Other: Sham laser
- Active Comparator: Laser treatment group

Laser treatment and sham injection at day 0 for 1st time, the investigators will decide whether the repeated laser treatment is needed according to monthly results during the visit after 3 months.

**Interventions:**

- - Other: Sham injection
  - Procedure: Laser

**Eligibility Criteria**

**Ages Eligible for Study:** 18 Years and older (Adult, Older Adult)

**Sexes Eligible for Study:** All

**Accepts Healthy Volunteers:** No

**Criteria:**

Inclusion Criteria:

1. Patients sign informed consent, and are willing and able to comply with all the follow-ups;
2. Age ≥ 18 years, both genders;
3. Diagnosis of type 1 or type 2 diabetes mellitus;
4. Serum HbA1c ≤ 10%;
5. Study eye must meet the following criteria:
   - Visual acuity impairment caused by DME with involving foveal;
   - BCVA score ≥ 24 and ≤ 73 Early Treatment Diabetic Retinopathy Study (ETDRS) letters at 4 meter/1 meter of ETDRS test (Equivalent Snellen chart 20/40 to 20/320);
   - Visual impairment due to Choroidal Neovascularization (CNV) secondary to high myopia.
   - Central retinal thickness (CRT) ≥300μm (spectral domain Optical Coherence Tomography (OCT), the CRT measurements must be confirmed by central reading center);
   - Refractive media opacities and miosis have no effect on the fundus examination.
6. Non-study eye BCVA ≥ 24 letters (equivalent to Snellen visual acuity 20/320).

Exclusion Criteria:

1. Active infectious ocular inflammation in either eye;
2. Proliferative diabetic retinopathy (PDR) in the study eye, with the exception of inactive, regressed PDR;
3. Any other ocular disorder in the study eye that may cause macular edema excluded the diabetic retinopathy;
4. Iris neovascularization in the study eye;
5. Uncontrolled glaucoma, or history of glaucoma surgery;
6. Aphakia in the study eye;
7. History of vitrectomy in the study eye;
8. History of panretinal laser photocoagulation (PRP) in the study eye 6 months prior to the screening, or possibly need panretinal photocoagulation of the study eye during the study;
9. Liver, kidney dysfunction;
10. History of allergic reaction to fluorescein, protein agents for diagnosis or therapy, or more than 2 drug or nondrug factors, or concomitant allergic diseases.

The SHINY Study

**Study Design**

**Study Type:** Interventional (Clinical Trial)

**Actual Enrollment:** 176 participants

**Allocation:** Randomized

**Intervention Model:** Parallel Assignment

**Masking:** Double (Participant, Investigator)

**Primary Purpose:** Treatment

**Intervention:**

- Drug: conbercept, Fixed (intravitreal injection of 0.5 mg conbercept per month, fixed injection)
- Drug: conbercept, PRN (intravitreal injection of 0.5 mg conbercept as need, PRN)
- Other: sham injection (sham intravitreal injection per month, fixed injection)

**Study Arms:**

- Experimental: conbercept treatment group

Subjects will receive conbercept injections at a dose of 0.5 mg/eye, once a month for first 3 months. In the next 6 months, the investigator will decide whether repeat injections are needed base on the monthly assessment results.

**Interventions:**

- - Drug: conbercept, Fixed
  - Drug: conbercept, PRN
- Sham Comparator: sham injection group

Subjects will receive sham injections monthly for 3 months and will receive 0.5 mg/eye conbercept at month 4. The investigator will decide whether repeat injections are needed base on the monthly assessment results from month 5 to month 9.

**Interventions:**

- - Drug: conbercept, Fixed
  - Drug: conbercept, PRN
  - Other: sham injection

**Eligibility Criteria**

**Ages Eligible for Study:** 18 Years and older (Adult, Older Adult)

**Sexes Eligible for Study:** All

**Accepts Healthy Volunteers:** No

**Criteria:**

Inclusion Criteria:

1. Patients give fully informed consent and are willing and able to comply with all study procedures.
2. In the study eye:
   - Myopia of equal to or greater than -6.00D, and axial length ≥26.5mm.
   - Diagnosis of active subfoveal, juxafoveal, or extrafoveal CNV secondary to high myopia.
   - Visual impairment due to CNV secondary to high myopia.
   - BCVA score ≥24 and ≤73 ETDRS letters (approximately 20/40~ 20/320 Snellen equivalent).
   - Ocular media of sufficient quality to obtain fundus and OCT images.
3. The BCVA score of fellow eyes ≥ 19 ETDRS letters (approximately 20/400 Snellen equivalent)

Exclusion Criteria:

1. Current vitreous hemorrhage in either eye.
2. Intraocular treatment with corticosteroids within last 3 months or periocular treatment with corticosteroids within last month in the study eye.
3. Active infectious ocular inflammation in either eye.
4. Fibrosis or atrophy involving the center of foveal in the study eye.
5. Any concurrent intraocular condition in the study eye that, in the opinion of investigator, could either require medical or surgical intervention during the study period to prevent or treat visual loss that might result from that condition.
6. The lesion size ≥30 mm2 in the study eye.
7. Any ocular disorder in the study eye that, in the opinion of the investigator, may confound interpretation of the study results.
8. Uncontrolled glaucoma or cup/disk ratio > 0.8 in the study eye.
9. Aphakia (excluding artificial lens) in the study eye.
10. Serious amblyopia and amaurosis in the fellow eye.

**Part B. Sensitivity Analysis**

**Supplemental Materials**

| **Table of Contents** | **Page** |
| --- | --- |
| **Table S1.** The fluctuation of the prices of conbercept and ranibizumab. | 1 |
| **Table S2.** The cost-effectiveness analyses of conbercept and ranibizumab with the fluctuation of prices. | 1 |
| **Table S3.** The one-way sensitivity analysis with different numbers of conbercept injections. | 3 |
| **Table S4.** The one-way sensitivity analysis with different numbers of ranibizumab injections. | 4 |
| **Table S5.** The two-way sensitivity analysis with different numbers of injections of conbercept and ranibizumab. | 5 |
| **Figure S1.** The two-way sensitivity analysis with different numbers of injections in treatment of AMD. | 9 |
| **Figure S2.** The two-way sensitivity analysis with different numbers of injections in treatment of DME. | 10 |
| **Figure S3.** The two-way sensitivity analysis with different numbers of injections in treatment of PM. | 11 |
| **Table S6.** The probabilistic sensitivity analysis of conbercept and ranibizumab. | 11 |
| **Figure S4.** The Cost-Effectiveness Acceptability Curve of conbercept and ranibizumab in treatment of AMD. | 13 |
| **Figure S5.** The Cost-Effectiveness Acceptability Curve of conbercept and ranibizumab in treatment of DME. | 14 |
| **Figure S6.** The Cost-Effectiveness Acceptability Curve of conbercept and ranibizumab in treatment of PM. | 15 |
| **Figure S7.** The Monte Carlo strategy selections of conbercept and ranibizumab in treatment of DME and PM. | 16 |
| **Table S7.** The cost-effectiveness analyses of conbercept and ranibizumab at the latest negotiated prices. | 17 |

**Part 1** Sensitivity analysis of the fluctuation of prices

**Table S1**. The fluctuation of the prices of conbercept and ranibizumab.

|  | Conbercept | Ranibizumab |
| --- | --- | --- |
| Original | 5550 | 5700 |
| up 5% | 5827.5 | 5985 |
| up 10% | 6105 | 6270 |
| down 5% | 5272.5 | 5415 |
| down 10% | 4995 | 5130 |

**Table S2**. The cost-effectiveness analyses of conbercept and ranibizumab with the fluctuation of prices.

|  | Price | Strategy | Cost (RMB) | Incremental costs | Effectiveness (QALYs) | Incremental effectiveness | CER | ICER |
| --- | --- | --- | --- | --- | --- | --- | --- | --- |
| Age-related macular degeneration | up 5% | conbercept | 296143.192 | -49958.355 | 7.825 | -0.665 | 37843.652 | 75209.322 |
|  |  | ranibizumab | 346101.547 | - | 8.490 | - | 40767.246 | - |
|  | up 10% | conbercept | 310245.249 | -52337.324 | 7.825 | -0.665 | 39645.730 | 78790.718 |
|  |  | ranibizumab | 362582.573 | - | 8.490 | - | 42708.543 | - |
|  | down 5% | conbercept | 267939.079 | -45200.416 | 7.825 | -0.665 | 34239.494 | 68046.530 |
|  |  | ranibizumab | 313139.495 | - | 8.490 | - | 36884.651 | - |
|  | down 10% | conbercept | 253837.022 | -42821.447 | 7.825 | -0.665 | 32437.416 | 64465.133 |
|  |  | ranibizumab | 296658.469 | - | 8.490 | - | 34943.354 | - |
| Diabetic macular edema | up 5% | conbercept | 338901.921 | -58473.930 | 6.973 | 0.215 | 46285.914 | -271754.592 |
|  |  | ranibizumab | 397375.850 | - | 6.758 | - | 56000.033 | - |
|  | up 10% | conbercept | 355040.108 | -61258.402 | 6.973 | 0.215 | 50914.505 | -284695.287 |
|  |  | ranibizumab | 416298.510 | - | 6.758 | - | 61600.037 | - |
|  | down 5% | conbercept | 306625.547 | -52904.984 | 6.973 | 0.215 | 43971.618 | -245873.202 |
|  |  | ranibizumab | 359530.531 | - | 6.758 | - | 53200.032 | - |
|  | down 10% | conbercept | 290487.361 | -50120.511 | 6.973 | 0.215 | 41657.322 | -232932.507 |
|  |  | ranibizumab | 340607.872 | - | 6.758 | - | 50400.030 | - |
| Pathological myopia | up 5% | conbercept | 111916.362 | -11141.966 | 7.528 | 0.029 | 14867.440 | -391844.667 |
|  |  | ranibizumab | 123058.328 | - | 7.499 | - | 16409.571 | - |
|  | up 10% | conbercept | 117245.712 | -11672.536 | 7.528 | 0.029 | 15575.414 | -410503.937 |
|  |  | ranibizumab | 128918.248 | - | 7.499 | - | 17190.979 | - |
|  | down 5% | conbercept | 101257.661 | -10080.827 | 7.528 | 0.029 | 13451.494 | -354526.127 |
|  |  | ranibizumab | 111338.487 | - | 7.499 | - | 14846.755 | - |
|  | down 10% | conbercept | 95928.310 | -9550.257 | 7.528 | 0.029 | 12743.520 | -335866.858 |
|  |  | ranibizumab | 105478.567 | - | 7.499 | - | 14065.347 | - |

**Part 2** Sensitivity analyses of different numbers of injections

**Table S3.** The one-way sensitivity analysis with different numbers of conbercept injections.

|  | Number of conbercept injections | Strategy | Cost (RMB) | Incremental costs | Effectiveness (QALYs) | Incremental effectiveness | CER | ICER |
| --- | --- | --- | --- | --- | --- | --- | --- | --- |
| AMD | 4.10 | Conbercept | 240910.137 | -88710.380 | 7.825 | -0.665 | 30785.510 | 133548.200 |
|  |  | Ranibizumab | 329620.521 | - | 8.490 | - | 38825.948 | - |
|  | 4.93 | Conbercept | 289385.957 | -40234.560 | 7.825 | -0.665 | 36980.156 | 60570.740 |
|  |  | Ranibizumab | 329620.521 | - | 8.490 | - | 38825.948 | - |
|  | 5.75 | Conbercept | 337861.777 | 8241.256 | 7.825 | -0.665 | 43174.801 | -12406.700 |
|  |  | Ranibizumab | 329620.521 | - | 8.490 | - | 38825.948 | - |
|  | 6.58 | Conbercept | 386337.597 | 56717.080 | 7.825 | -0.665 | 49369.447 | -85384.200 |
|  |  | Ranibizumab | 329620.521 | - | 8.490 | - | 38825.948 | - |
|  | 7.40 | Conbercept | 434813.417 | 105192.900 | 7.825 | -0.665 | 55564.092 | -158362.000 |
|  |  | Ranibizumab | 329620.521 | - | 8.490 | - | 38825.948 | - |
| DME | 5.30 | Conbercept | 311026.871 | -67426.320 | 6.973 | 0.215 | 44602.790 | -313360.367 |
|  |  | Ranibizumab | 378453.191 | - | 6.758 | - | 56000.033 | - |
|  | 5.70 | Conbercept | 334500.597 | -43952.594 | 6.973 | 0.215 | 47969.038 | -204267.427 |
|  |  | Ranibizumab | 378453.191 | - | 6.758 | - | 56000.033 | - |
|  | 6.10 | Conbercept | 357974.323 | -20478.868 | 6.973 | 0.215 | 51335.286 | -95174.488 |
|  |  | Ranibizumab | 378453.191 | - | 6.758 | - | 56000.033 | - |
|  | 6.50 | Conbercept | 381448.049 | 2994.859 | 6.973 | 0.215 | 54701.535 | 13918.452 |
|  |  | Ranibizumab | 378453.191 | - | 6.758 | - | 56000.033 | - |
| PM | 1.80 | Conbercept | 106587.011 | -10611.396 | 7.528 | 0.029 | 14159.467 | 373185.397 |
|  |  | Ranibizumab | 117198.408 | - | 7.499 | - | 15628.163 | - |
|  | 2.34 | Conbercept | 138267.039 | 21068.632 | 7.528 | 0.029 | 18367.975 | 740949.207 |
|  |  | Ranibizumab | 117198.408 | - | 7.499 | - | 15628.163 | - |
|  | 2.87 | Conbercept | 169947.068 | 52748.660 | 7.528 | 0.029 | 22576.484 | 1855083.812 |
|  |  | Ranibizumab | 117198.408 | - | 7.499 | - | 15628.163 | - |
|  | 3.41 | Conbercept | 201627.096 | 84428.688 | 7.528 | 0.029 | 26784.992 | 2969218.417 |
|  |  | Ranibizumab | 117198.408 | - | 7.499 | - | 15628.163 | - |
|  | 3.94 | Conbercept | 233307.124 | 116108.717 | 7.528 | 0.029 | 30993.500 | 4083353.021 |
|  |  | Ranibizumab | 117198.408 | - | 7.499 | - | 15628.163 | - |

**Table S4.** The one-way sensitivity analysis with different numbers of ranibizumab injections.

|  | Number of ranibizumab injections | Strategy | Cost (RMB) | Incremental costs | Effectiveness (QALYs) | Incremental effectiveness | CER | ICER |
| --- | --- | --- | --- | --- | --- | --- | --- | --- |
| AMD | 4.70 | Conbercept | 282041.136 | -4850.799 | 7.825 | -0.665 | 36041.573 | 7302.589 |
|  |  | Ranibizumab | 286891.935 | - | 8.490 | - | 33792.955 | - |
|  | 5.70 | Conbercept | 282041.136 | -65891.637 | 7.825 | -0.665 | 36041.573 | 99195.928 |
|  |  | Ranibizumab | 347932.772 | - | 8.490 | - | 40982.946 | - |
|  | 6.70 | Conbercept | 282041.136 | -126932.474 | 7.825 | -0.665 | 36041.573 | 191089.266 |
|  |  | Ranibizumab | 408973.610 | - | 8.490 | - | 48172.936 | - |
|  | 7.70 | Conbercept | 282041.136 | -187973.311 | 7.825 | -0.665 | 36041.573 | 282982.605 |
|  |  | Ranibizumab | 470014.447 | - | 8.490 | - | 55362.926 | - |
|  | 8.70 | Conbercept | 282041.136 | -249014.148 | 7.825 | -0.665 | 36041.573 | 374875.943 |
|  |  | Ranibizumab | 531055.284 | - | 8.490 | - | 62552.917 | - |
| DME | 4.10 | Conbercept | 322763.734 | 72496.301 | 6.973 | 0.215 | 46285.914 | 336922.846 |
|  |  | Ranibizumab | 250267.433 | - | 6.758 | - | 37032.280 | - |
|  | 5.13 | Conbercept | 322763.734 | 9420.770 | 6.973 | 0.215 | 46285.914 | 43782.544 |
|  |  | Ranibizumab | 313342.964 | - | 6.758 | - | 46365.619 | - |
|  | 6.17 | Conbercept | 322763.734 | -53654.762 | 6.973 | 0.215 | 46285.914 | -249357.758 |
|  |  | Ranibizumab | 376418.496 | - | 6.758 | - | 55698.958 | - |
|  | 7.20 | Conbercept | 322763.734 | -116730.294 | 6.973 | 0.215 | 46285.914 | -542498.061 |
|  |  | Ranibizumab | 439494.028 | - | 6.758 | - | 65032.297 | - |
| PM | 1.92 | Conbercept | 106587.011 | -10611.396 | 7.528 | 0.029 | 14159.467 | 373185.397 |
|  |  | Ranibizumab | 117198.408 | - | 7.499 | - | 15628.163 | - |
|  | 2.46 | Conbercept | 106587.011 | -43268.244 | 7.528 | 0.029 | 14159.467 | 1521673.145 |
|  |  | Ranibizumab | 149855.255 | - | 7.499 | - | 19982.886 | - |
|  | 2.99 | Conbercept | 106587.011 | -75925.092 | 7.528 | 0.029 | 14159.467 | 2670160.893 |
|  |  | Ranibizumab | 182512.103 | - | 7.499 | - | 24337.608 | - |
|  | 3.53 | Conbercept | 106587.011 | -108581.940 | 7.528 | 0.029 | 14159.467 | 3818648.641 |
|  |  | Ranibizumab | 215168.951 | - | 7.499 | - | 28692.331 | - |
|  | 4.06 | Conbercept | 106587.011 | -141238.788 | 7.528 | 0.029 | 14159.467 | 4967136.389 |
|  |  | Ranibizumab | 247825.799 | - | 7.499 | - | 33047.053 | - |

| **Table S5.** The two-way sensitivity analysis with different numbers of injections of conbercept and ranibizumab. | | | | | | | | | | | |
| --- | --- | --- | --- | --- | --- | --- | --- | --- | --- | --- | --- |
|  | Conbercept | Ranibizumab | Cost (RMB)-C | Cost (RMB)-R | Incremental costs | Effectiveness (QALYs)-C | Effectiveness (QALYs)-R | Incremental effectiveness | CER-C | CER-R | ICER |
| AMD | 4.10 | 4.70 | 240910.137 | 286891.935 | -45981.798 | 7.825 | 8.490 | -0.664 | 30785.510 | 33792.955 | 69222.854 |
|  | 4.10 | 5.70 | 240910.137 | 347932.772 | -107022.636 | 7.825 | 8.490 | -0.664 | 30785.510 | 40982.946 | 161116.192 |
|  | 4.10 | 6.70 | 240910.137 | 408973.610 | -168063.473 | 7.825 | 8.490 | -0.664 | 30785.510 | 48172.936 | 253009.531 |
|  | 4.10 | 7.70 | 240910.137 | 470014.447 | -229104.310 | 7.825 | 8.490 | -0.664 | 30785.510 | 55362.926 | 344902.869 |
|  | 4.10 | 8.70 | 240910.137 | 531055.284 | -290145.147 | 7.825 | 8.490 | -0.664 | 30785.510 | 62552.917 | 436796.208 |
|  | 4.93 | 4.70 | 289385.957 | 286891.935 | 2494.022 | 7.825 | 8.490 | -0.664 | 36980.156 | 33792.955 | -3754.601 |
|  | 4.93 | 5.70 | 289385.957 | 347932.772 | -58546.815 | 7.825 | 8.490 | -0.664 | 36980.156 | 40982.946 | 88138.737 |
|  | 4.93 | 6.70 | 289385.957 | 408973.610 | -119587.653 | 7.825 | 8.490 | -0.664 | 36980.156 | 48172.936 | 180032.076 |
|  | 4.93 | 7.70 | 289385.957 | 470014.447 | -180628.490 | 7.825 | 8.490 | -0.664 | 36980.156 | 55362.926 | 271925.414 |
|  | 4.93 | 8.70 | 289385.957 | 531055.284 | -241669.327 | 7.825 | 8.490 | -0.664 | 36980.156 | 62552.917 | 363818.753 |
|  | 5.75 | 4.70 | 337861.777 | 286891.935 | 50969.842 | 7.825 | 8.490 | -0.664 | 43174.801 | 33792.955 | -76732.056 |
|  | 5.75 | 5.70 | 337861.777 | 347932.772 | -10070.995 | 7.825 | 8.490 | -0.664 | 43174.801 | 40982.946 | 15161.282 |
|  | 5.75 | 6.70 | 337861.777 | 408973.610 | -71111.832 | 7.825 | 8.490 | -0.664 | 43174.801 | 48172.936 | 107054.621 |
|  | 5.75 | 7.70 | 337861.777 | 470014.447 | -132152.670 | 7.825 | 8.490 | -0.664 | 43174.801 | 55362.926 | 198947.959 |
|  | 5.75 | 8.70 | 337861.777 | 531055.284 | -193193.507 | 7.825 | 8.490 | -0.664 | 43174.801 | 62552.917 | 290841.298 |
|  | 6.58 | 4.70 | 386337.597 | 286891.935 | 99445.662 | 7.825 | 8.490 | -0.664 | 49369.447 | 33792.955 | -149709.511 |
|  | 6.58 | 5.70 | 386337.597 | 347932.772 | 38404.825 | 7.825 | 8.490 | -0.664 | 49369.447 | 40982.946 | -57816.172 |
|  | 6.58 | 6.70 | 386337.597 | 408973.610 | -22636.012 | 7.825 | 8.490 | -0.664 | 49369.447 | 48172.936 | 34077.166 |
|  | 6.58 | 7.70 | 386337.597 | 470014.447 | -83676.850 | 7.825 | 8.490 | -0.664 | 49369.447 | 55362.926 | 125970.504 |
|  | 6.58 | 8.70 | 386337.597 | 531055.284 | -144717.687 | 7.825 | 8.490 | -0.664 | 49369.447 | 62552.917 | 217863.843 |
|  | 7.40 | 4.70 | 434813.417 | 286891.935 | 147921.482 | 7.825 | 8.490 | -0.664 | 55564.092 | 33792.955 | -222686.966 |
|  | 7.40 | 5.70 | 434813.417 | 347932.772 | 86880.645 | 7.825 | 8.490 | -0.664 | 55564.092 | 40982.946 | -130793.627 |
|  | 7.40 | 6.70 | 434813.417 | 408973.610 | 25839.808 | 7.825 | 8.490 | -0.664 | 55564.092 | 48172.936 | -38900.289 |
|  | 7.40 | 7.70 | 434813.417 | 470014.447 | -35201.029 | 7.825 | 8.490 | -0.664 | 55564.092 | 55362.926 | 52993.049 |
|  | 7.40 | 8.70 | 434813.417 | 531055.284 | -96241.867 | 7.825 | 8.490 | -0.664 | 55564.092 | 62552.917 | 144886.388 |
| DME | 5.30 | 4.10 | 311026.871 | 250267.433 | 60759.438 | 6.973 | 6.758 | 0.215 | 44602.790 | 37032.280 | 282376.377 |
|  | 5.30 | 5.13 | 311026.871 | 313342.964 | -2316.093 | 6.973 | 6.758 | 0.215 | 44602.790 | 46365.619 | -10763.926 |
|  | 5.30 | 6.17 | 311026.871 | 376418.496 | -65391.625 | 6.973 | 6.758 | 0.215 | 44602.790 | 55698.958 | -303904.228 |
|  | 5.30 | 7.20 | 311026.871 | 439494.028 | -128467.157 | 6.973 | 6.758 | 0.215 | 44602.790 | 65032.297 | -597044.530 |
|  | 5.70 | 4.10 | 334500.597 | 250267.433 | 84233.164 | 6.973 | 6.758 | 0.215 | 47969.038 | 37032.280 | 391469.316 |
|  | 5.70 | 5.13 | 334500.597 | 313342.964 | 21157.633 | 6.973 | 6.758 | 0.215 | 47969.038 | 46365.619 | 98329.014 |
|  | 5.70 | 6.17 | 334500.597 | 376418.496 | -41917.899 | 6.973 | 6.758 | 0.215 | 47969.038 | 55698.958 | -194811.289 |
|  | 5.70 | 7.20 | 334500.597 | 439494.028 | -104993.431 | 6.973 | 6.758 | 0.215 | 47969.038 | 65032.297 | -487951.591 |
|  | 6.10 | 4.10 | 357974.323 | 250267.433 | 107706.891 | 6.973 | 6.758 | 0.215 | 51335.286 | 37032.280 | 500562.255 |
|  | 6.10 | 5.13 | 357974.323 | 313342.964 | 44631.359 | 6.973 | 6.758 | 0.215 | 51335.286 | 46365.619 | 207421.953 |
|  | 6.10 | 6.17 | 357974.323 | 376418.496 | -18444.173 | 6.973 | 6.758 | 0.215 | 51335.286 | 55698.958 | -85718.349 |
|  | 6.10 | 7.20 | 357974.323 | 439494.028 | -81519.705 | 6.973 | 6.758 | 0.215 | 51335.286 | 65032.297 | -378858.651 |
|  | 6.50 | 4.10 | 381448.049 | 250267.433 | 131180.617 | 6.973 | 6.758 | 0.215 | 54701.535 | 37032.280 | 609655.195 |
|  | 6.50 | 5.13 | 381448.049 | 313342.964 | 68105.085 | 6.973 | 6.758 | 0.215 | 54701.535 | 46365.619 | 316514.893 |
|  | 6.50 | 6.17 | 381448.049 | 376418.496 | 5029.553 | 6.973 | 6.758 | 0.215 | 54701.535 | 55698.958 | 23374.590 |
|  | 6.50 | 7.20 | 381448.049 | 439494.028 | -58045.979 | 6.973 | 6.758 | 0.215 | 54701.535 | 65032.297 | -269765.712 |
| PM | 1.80 | 1.92 | 106587.011 | 117198.407 | -10611.396 | 7.528 | 7.499 | 0.028 | 14159.467 | 15628.163 | -373185.397 |
|  | 1.80 | 2.46 | 106587.011 | 149855.255 | -43268.244 | 7.528 | 7.499 | 0.028 | 14159.467 | 19982.886 | -1521673.145 |
|  | 1.80 | 2.99 | 106587.011 | 182512.103 | -75925.092 | 7.528 | 7.499 | 0.028 | 14159.467 | 24337.608 | -2670160.893 |
|  | 1.80 | 3.52 | 106587.011 | 215168.951 | -108581.940 | 7.528 | 7.499 | 0.028 | 14159.467 | 28692.331 | -3818648.641 |
|  | 1.80 | 4.06 | 106587.011 | 247825.799 | -141238.788 | 7.528 | 7.499 | 0.028 | 14159.467 | 33047.053 | -4967136.389 |
|  | 2.34 | 1.92 | 138267.039 | 117198.407 | 21068.632 | 7.528 | 7.499 | 0.028 | 18367.975 | 15628.163 | 740949.207 |
|  | 2.34 | 2.46 | 138267.039 | 149855.255 | -11588.216 | 7.528 | 7.499 | 0.028 | 18367.975 | 19982.886 | -407538.540 |
|  | 2.34 | 2.99 | 138267.039 | 182512.103 | -44245.064 | 7.528 | 7.499 | 0.028 | 18367.975 | 24337.608 | -1556026.288 |
|  | 2.34 | 3.52 | 138267.039 | 215168.951 | -76901.912 | 7.528 | 7.499 | 0.028 | 18367.975 | 28692.331 | -2704514.036 |
|  | 2.34 | 4.06 | 138267.039 | 247825.799 | -109558.760 | 7.528 | 7.499 | 0.028 | 18367.975 | 33047.053 | -3853001.784 |
|  | 2.87 | 1.92 | 169947.068 | 117198.407 | 52748.660 | 7.528 | 7.499 | 0.028 | 22576.484 | 15628.163 | 1855083.812 |
|  | 2.87 | 2.46 | 169947.068 | 149855.255 | 20091.812 | 7.528 | 7.499 | 0.028 | 22576.484 | 19982.886 | 706596.064 |
|  | 2.87 | 2.99 | 169947.068 | 182512.103 | -12565.036 | 7.528 | 7.499 | 0.028 | 22576.484 | 24337.608 | -441891.684 |
|  | 2.87 | 3.52 | 169947.068 | 215168.951 | -45221.884 | 7.528 | 7.499 | 0.028 | 22576.484 | 28692.331 | -1590379.432 |
|  | 2.87 | 4.06 | 169947.068 | 247825.799 | -77878.732 | 7.528 | 7.499 | 0.028 | 22576.484 | 33047.053 | -2738867.179 |
|  | 3.41 | 1.92 | 201627.096 | 117198.407 | 84428.688 | 7.528 | 7.499 | 0.028 | 26784.992 | 15628.163 | 2969218.417 |
|  | 3.41 | 2.46 | 201627.096 | 149855.255 | 51771.841 | 7.528 | 7.499 | 0.028 | 26784.992 | 19982.886 | 1820730.669 |
|  | 3.41 | 2.99 | 201627.096 | 182512.103 | 19114.993 | 7.528 | 7.499 | 0.028 | 26784.992 | 24337.608 | 672242.921 |
|  | 3.41 | 3.52 | 201627.096 | 215168.951 | -13541.855 | 7.528 | 7.499 | 0.028 | 26784.992 | 28692.331 | -476244.827 |
|  | 3.41 | 4.06 | 201627.096 | 247825.799 | -46198.703 | 7.528 | 7.499 | 0.028 | 26784.992 | 33047.053 | -1624732.575 |
|  | 3.94 | 1.92 | 233307.124 | 117198.407 | 116108.717 | 7.528 | 7.499 | 0.028 | 30993.500 | 15628.163 | 4083353.021 |
|  | 3.94 | 2.46 | 233307.124 | 149855.255 | 83451.869 | 7.528 | 7.499 | 0.028 | 30993.500 | 19982.886 | 2934865.273 |
|  | 3.94 | 2.99 | 233307.124 | 182512.103 | 50795.021 | 7.528 | 7.499 | 0.028 | 30993.500 | 24337.608 | 1786377.526 |
|  | 3.94 | 3.52 | 233307.124 | 215168.951 | 18138.173 | 7.528 | 7.499 | 0.028 | 30993.500 | 28692.331 | 637889.778 |
|  | 3.94 | 4.06 | 233307.124 | 247825.799 | -14518.675 | 7.528 | 7.499 | 0.028 | 30993.500 | 33047.053 | -510597.970 |


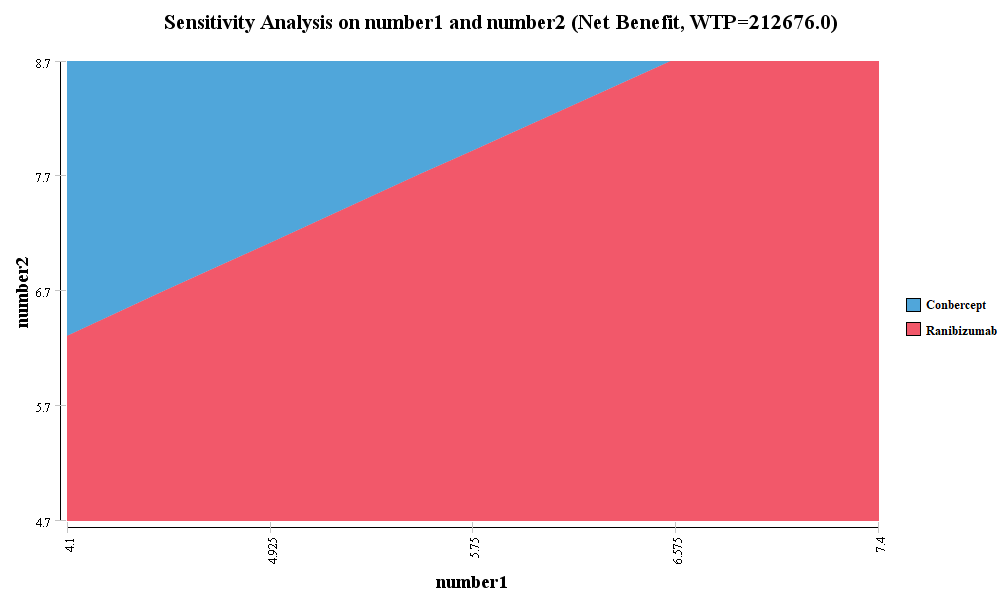


**Figure S1.** The two-way sensitivity analysis with different numbers of injections in treatment of AMD.


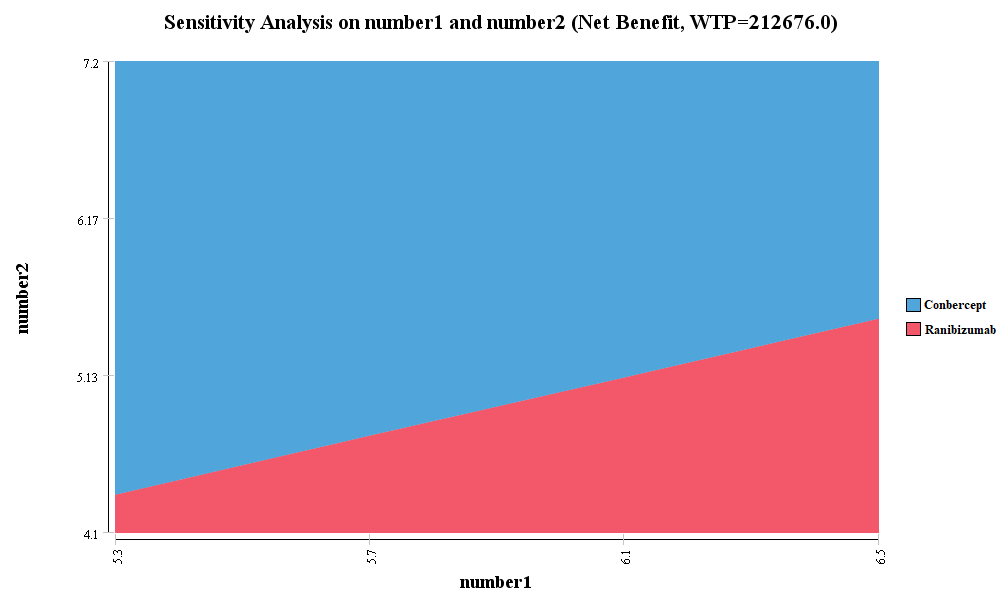


**Figure S2.** The two-way sensitivity analysis with different numbers of injections in treatment of DME.


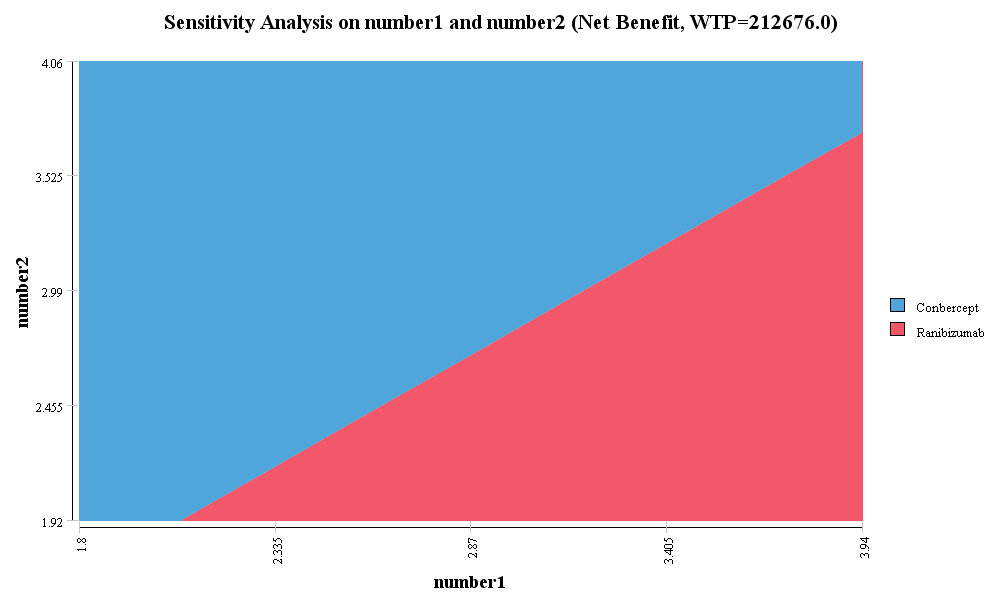


**Figure S3.** The two-way sensitivity analysis with different numbers of injections in treatment of PM.

| **Table S6.** The probabilistic sensitivity analysis of conbercept and ranibizumab. | | | | | | | | | | |
| --- | --- | --- | --- | --- | --- | --- | --- | --- | --- | --- |
|  | Statistic | Cost (RMB)-C | Cost (RMB)-R | Incremental costs | Effectiveness (QALYs)-C | Effectiveness (QALYs)-R | Incremental effectiveness | CER-C | CER-R | ICER |
| AMD | Mean | 285503.305 | 329888.704 | -44385.399 | 7.825 | 8.490 | -0.664 | 36486.045 | 38856.149 | 66845.480 |
|  | Minimum | 232078.319 | 277207.161 | -45128.842 | 7.825 | 8.490 | -0.664 | 29658.571 | 32651.020 | 67965.123 |
|  | *P*_2.5_ | 250753.657 | 298087.100 | -47333.443 | 7.825 | 8.490 | -0.664 | 32045.196 | 35110.377 | 71285.306 |
|  | *P*_10_ | 262033.810 | 307446.503 | -45412.693 | 7.825 | 8.490 | -0.664 | 33486.749 | 36212.780 | 68392.610 |
|  | Median | 284910.461 | 327231.963 | -42321.502 | 7.825 | 8.490 | -0.664 | 36410.283 | 38543.223 | 63737.202 |
|  | *P*_90_ | 308745.345 | 355726.169 | -46980.824 | 7.825 | 8.490 | -0.664 | 39456.274 | 41899.431 | 70754.253 |
|  | *P*_97.5_ | 322050.641 | 368122.275 | -46071.634 | 7.825 | 8.490 | -0.664 | 41156.631 | 43359.514 | 69384.991 |
|  | Maximum | 362736.455 | 385467.953 | -22731.498 | 7.825 | 8.490 | -0.664 | 46356.096 | 45402.586 | 34234.184 |
| DME | Mean | 325862.525 | 377275.646 | -51413.121 | 6.973 | 6.758 | 0.215 | 46732.041 | 55826.524 | -239130.795 |
|  | Minimum | 164378.071 | 195311.179 | -30933.108 | 6.973 | 6.758 | 0.215 | 23573.508 | 28900.737 | -143874.921 |
|  | *P*_2.5_ | 215372.964 | 268466.610 | -53093.646 | 6.973 | 6.758 | 0.215 | 30886.701 | 39725.749 | -246947.191 |
|  | *P*_10_ | 252341.053 | 303768.407 | -51427.354 | 6.973 | 6.758 | 0.215 | 36188.305 | 44949.454 | -239196.995 |
|  | Median | 323514.926 | 372360.927 | -48846.001 | 6.973 | 6.758 | 0.215 | 46395.372 | 55099.279 | -227190.702 |
|  | *P*_90_ | 403499.229 | 454615.666 | -51116.437 | 6.973 | 6.758 | 0.215 | 57865.944 | 67270.741 | -237750.870 |
|  | *P*_97.5_ | 440098.036 | 498012.402 | -57914.366 | 6.973 | 6.758 | 0.215 | 63114.590 | 73692.276 | -269369.144 |
|  | Maximum | 518945.312 | 563533.752 | -44588.440 | 6.973 | 6.758 | 0.215 | 74422.101 | 83387.652 | -207388.093 |
| PM | Mean | 177605.460 | 179611.273 | -2005.813 | 7.528 | 7.499 | 0.028 | 23592.649 | 23951.363 | -71636.179 |
|  | Minimum | 51279.223 | 51821.963 | -542.740 | 7.528 | 7.499 | 0.028 | 6811.799 | 6910.516 | -19383.571 |
|  | *P*_2.5_ | 82147.548 | 79263.279 | 2884.269 | 7.528 | 7.499 | 0.028 | 10912.267 | 10569.847 | 103009.607 |
|  | *P*_10_ | 104752.140 | 106166.997 | -1414.857 | 7.528 | 7.499 | 0.028 | 13915.003 | 14157.487 | -50530.607 |
|  | Median | 170429.076 | 166660.987 | 3768.089 | 7.528 | 7.499 | 0.028 | 22639.357 | 22224.428 | 134574.607 |
|  | *P*_90_ | 251010.357 | 263997.413 | -12987.056 | 7.528 | 7.499 | 0.028 | 33343.565 | 35204.349 | -463823.429 |
|  | *P*_97.5_ | 302664.468 | 341180.157 | -38515.689 | 7.528 | 7.499 | 0.028 | 40205.163 | 45496.754 | -1375560.321 |
|  | Maximum | 408112.714 | 466925.387 | -58812.673 | 7.528 | 7.499 | 0.028 | 54212.635 | 62265.020 | -2100452.607 |


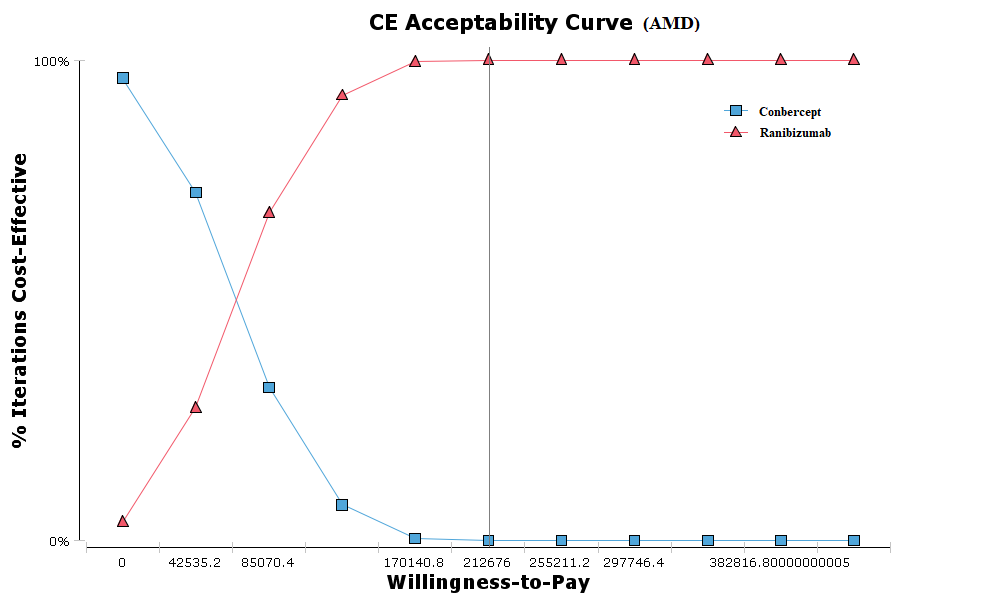


**Figure S4.** The Cost-Effectiveness Acceptability Curve of conbercept and ranibizumab in treatment of AMD.


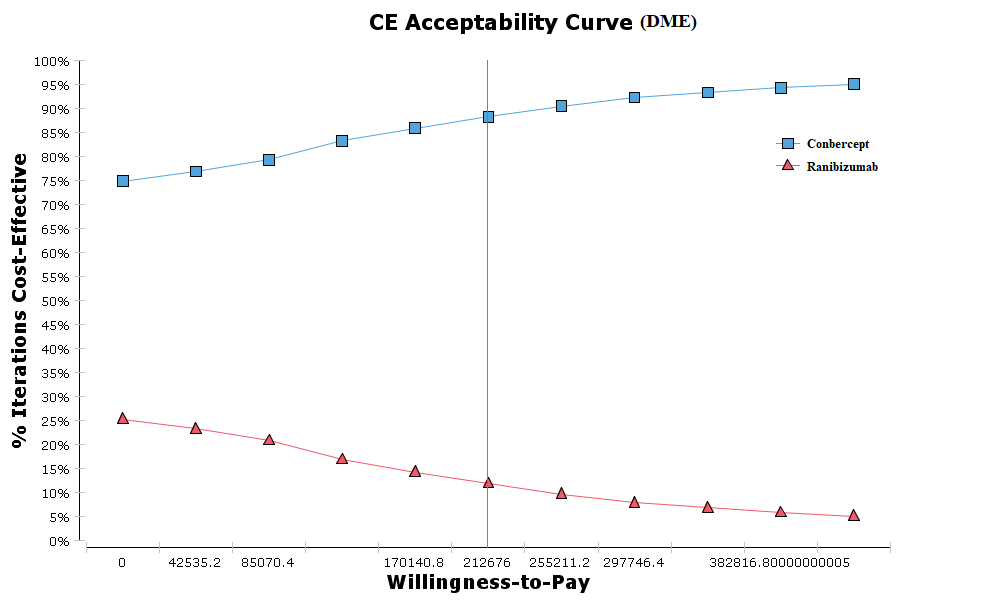


**Figure S5.** The Cost-Effectiveness Acceptability Curve of conbercept and ranibizumab in treatment of DME.


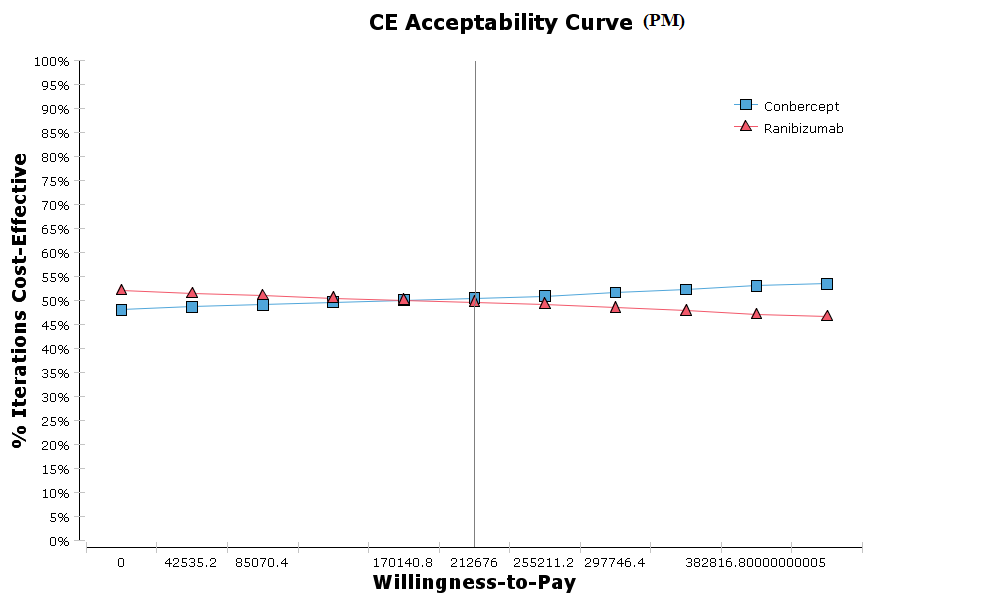


**Figure S6.** The Cost-Effectiveness Acceptability Curve of conbercept and ranibizumab in treatment of PM.


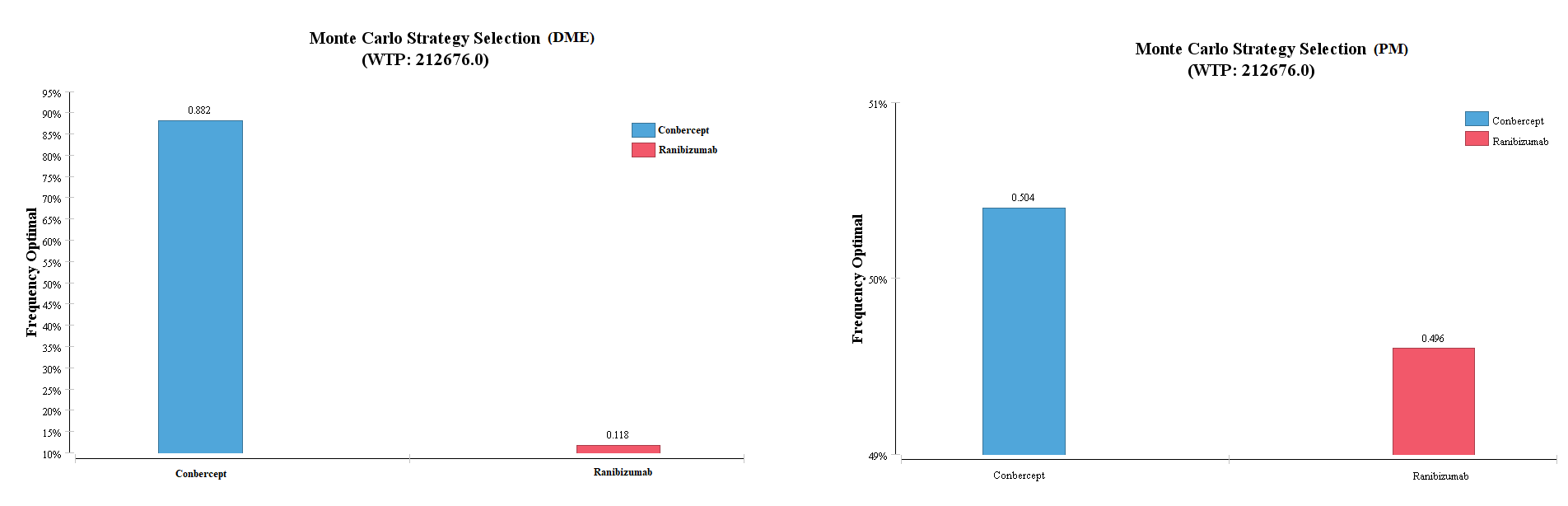


**Figure S7.** The Monte Carlo strategy selections of conbercept and ranibizumab in treatment of DME and PM.

**Part 3** Sensitivity analyses of the latest negotiated prices

**Table S7.** The cost-effectiveness analyses of conbercept and ranibizumab at the latest negotiated prices.

|  | Strategy | Cost (RMB) | Incremental costs | Effectiveness (QALYs) | Incremental effectiveness | CER | ICER |
| --- | --- | --- | --- | --- | --- | --- | --- |
| Age-related macular degeneration | conbercept | 213835.691 | -14585.547 | 7.825 | -0.665 | 27325.711 | 21957.671 |
|  | ranibizumab | 228421.238 | - | 8.490 | - | 26905.701 | - |
| Diabetic macular edema | conbercept | 241927.412 | -20334.010 | 6.973 | 0.215 | 34693.586 | -94501.271 |
|  | ranibizumab | 262261.422 | - | 6.758 | - | 38807.041 | - |
| Pathological myopia | conbercept | 79892.246 | -1324.194 | 7.528 | 0.029 | 10613.222 | -46569.735 |
|  | ranibizumab | 81216.440 | - | 7.499 | - | 10830.043 | - |
